# Investigation of the Brain Biodistribution of the Lipoprotein-Associated Phospholipase A_2_ (Lp-PLA_2_) Inhibitor [^18^F]GSK2647544 in Healthy Male Subjects

**DOI:** 10.1007/s11307-016-0982-5

**Published:** 2016-07-11

**Authors:** Mickael Huiban, Christopher Coello, Kai Wu, Yanmei Xu, Yvonne Lewis, Andrew P. Brown, Mauro Buraglio, Chenbing Guan, Shaila Shabbir, Regan Fong, Jan Passchier, Eugenii A. Rabiner, Andrew Lockhart

**Affiliations:** 1 Imanova Limited, Burlington Danes Building, Imperial College London, Hammersmith Hospital, Du Cane Road, London, W12 0NN UK; 2 WuXi Clinical Development Service, 19th Floor, Building A, FuXing Plaza, 388 Ma Dang Road, Shanghai, 200025 China; 3 GlaxoSmithKline, Neurosciences, 917 Halei Road, Zhangjiang Hi-tech Park, Pudong, Shanghai 201203 China; 4 AstraZeneca UK Limited, Melbourn Science Park, Royston, Herts SG8 6HB UK; 5 GlaxoSmithKline, Neurosciences, Gunnels Wood Road, Stevenage, SG1 2NY UK; 6 UCB Biosciences Inc, PO Box 110167, Research Triangle Park, NC 27709 USA; 7 GlaxoSmithKline, Neurosciences, Clinical Unit Cambridge, Addenbrooke’s Hospital, Cambridge, CB2 0GG UK

**Keywords:** Lp-PLA_2_, PET, Alzheimer’s disease, GSK2647544, Biodistribution, Positron emission tomography

## Abstract

**Purpose:**

GSK2647544 is a potent and specific inhibitor of lipoprotein-associated phospholipase A_2_ (Lp-PLA_2_), which was in development as a potential treatment for Alzheimer’s disease (AD). In order to refine therapeutic dose predictions and confirm brain penetration, a radiolabelled form of the inhibitor, [^18^F]GSK2647544, was manufactured for use in a positron emission tomography (PET) biodistribution study.

**Procedures:**

[^18^F]GSK2647544 was produced using a novel, copper iodide (Cu(I)) mediated, [^18^F]trifluoromethylation methodology. Healthy male subjects (*n* = 4, age range 34–42) received an oral dose of unlabelled GSK2647544 (100 mg) and after 2 h an intravenous (iv) injection of [^18^F]GSK2647544 (average injected activity and mass were 106 ± 47 MBq and 179 ± 55 μg, respectively) followed by dynamic PET scans for 120 min. Defined regions of interest (ROI) throughout the brain were used to obtain regional time-activity curves (TACs) and compartmental modelling analysis used to estimate the primary outcome measure, whole brain volume of distribution (V_T_). Secondary PK and safety endpoints were also recorded.

**Results:**

PET dynamic data were successfully obtained from all four subjects and there were no clinically significant variations of the safety endpoints. Inspection of the TACs indicated a relatively homogenous uptake of [^18^F]GSK2647544 across all the ROIs examined. The mean whole brain V_T_ was 0.56 (95 % CI, 0.41–0.72). Secondary PK parameters, C_max_ (geometric mean) and T_max_ (median), were 354 ng/ml and 1.4 h, respectively. Metabolism of GSK2647544 was relatively consistent across subjects, with 20–40 % of the parent compound [^18^F]GSK2647544 present after 120 min.

**Conclusions:**

The study provides evidence that GSK2647544 is able to cross the blood brain barrier in healthy male subjects leading to a measurable brain exposure. The administered doses of GSK2647544 were well tolerated. Exploratory modelling suggested that a twice-daily dose of 102 mg, at steady state, would provide ~80 % trough inhibition of brain Lp-PLA_2_ activity.

**Trial Registration:**

Clintrials.gov: NCT01924858.

**Electronic supplementary material:**

The online version of this article (doi:10.1007/s11307-016-0982-5) contains supplementary material, which is available to authorized users.

## Introduction

Alzheimer’s disease (AD) is a progressive neurodegenerative disorder that accounts for ~60–70 % of >47.5 million people affected worldwide by dementia [1]. The disease is characterised neuropathologically by the formation of amyloid-beta containing senile plaques and tau containing neurofibrillary tangles and clinically by cognitive deficits that over time result in profound cognitive and functional impairment [2]. AD remains an area of high unmet medical need as currently available treatment options for AD are limited and provide only modest, short-term symptomatic benefit [3].

Lipoprotein-associated phospholipase A2 (Lp-PLA_2_), also known as plasma platelet activating factor acetylhydrolase (PAF-AH), is a member of the phospholipase A_2_ superfamily of enzymes [4]. Lp-PLA_2_ is primarily secreted by monocyte-derived macrophages and its expression is upregulated during inflammation [5]. Lp-PLA_2_ circulates in plasma as a complex with low-density lipoprotein (LDL) and, to a lesser extent, with high-density lipoprotein (HDL) and lipoprotein(a) [5, 6]. Lp-PLA_2_ has been studied extensively as a marker of cardiovascular risk, and inhibitors of the enzyme were initially developed to counter the role of products derived from oxidised low-density lipoproteins in driving the vascular inflammation [6].

Preclinical evidence suggested beneficial effects of Lp-PLA_2_ inhibition on blood brain permeability and brain amyloid-beta peptide 1-42 (Aβ1-42) deposition [7]. Preliminary clinical evidence that targeting this pathway may provide a novel treatment to slow the progression of AD comes from a Phase 2a study with the Lp-PLA_2_ inhibitor, rilapladib (SB659032) [8]. Lp-PLA_2_ is also present in the human central nervous system (CNS) [9], and its specific activity can be detected in cerebrospinal fluid [8, 10]. Pharmacological inhibition of this central pool of Lp-PLA_2_, in addition to the plasma pool, has the potential to provide an additional interventional mechanism for the treatment of AD and other neurological indications.

**Fig. 1:**
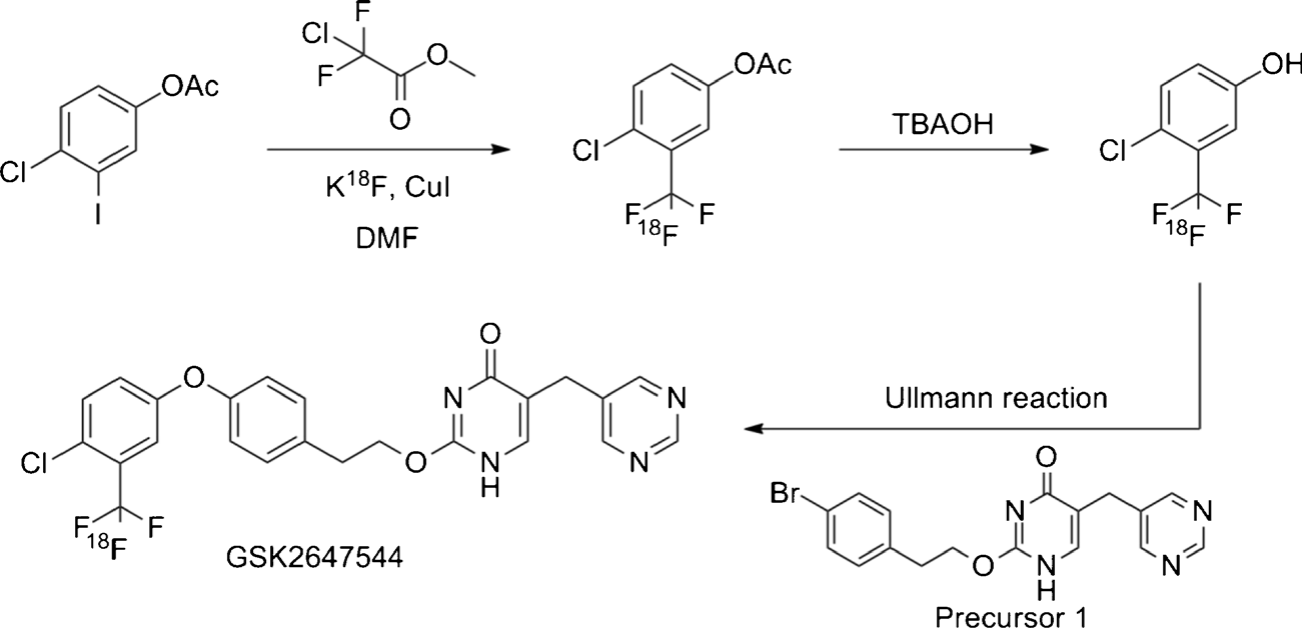
Multi-step approach to the preparation of [^18^F]GSK2647544 using the methodology described in [12]. A bromide-containing precursor, 2-(4-bromophenethoxy)-5-(pyrimidin-5-ylmethyl)pyrimidin-4(1H)-one,was coupled to the 4-chloro-3-([^18^F]trifluoromethyl)phenol reaction product to generate [^18^F]GSK2647544.

GSK2647544 is a novel potent and specific inhibitor of Lp-PLA_2_, and data indicated that the molecule was able to cross the BBB of preclinical species [GSK data on file]. At the time of initiating the PET study, GSK2647544 had successfully completed a first time in human (FTIH) clinical trial (Clinicaltrials.gov: NCT01702467) [11]. In order to enable the PET biodistribution study, options for radiolabelling of GSK2647544 had to be identified. The chemical structure of the drug presented a significant challenge to conventional radiolabelling approaches due to the lack of functional groups amenable to existing PET chemistry approaches. Recently published work suggested the potential to insert the PET isotope [^18^F]fluoride in the trifluoromethyl position on the molecule [12] and a three-step synthesis was developed (Fig. 1), which was successfully implemented, optimised and transferred to a fully automated setup. Subsequently, the process was fully validated for clinical use.

The primary objective of the study was to investigate the brain penetration of GSK2647544 through determination of the whole brain PET volume of distribution (V_T_) [13] of [^18^F]GSK2647544, following administration of an oral dose of the unlabelled drug (100 mg) 2 h prior to the PET scan. Secondary endpoints included safety measures and PK parameters. An exploratory analysis was also performed to estimate the dosing regimen required to achieve, at trough, a level of ~80 % brain Lp-PLA_2_ inhibition. After 4 subjects had successfully completed the study, a protocol amendment was undertaken to permit an evaluation of the brain penetration of [^18^F]GSK2647544 in the absence of the oral dose of GSK2647544. The amended protocol was not initiated as the PET study was terminated due to a finding from a separate clinical pharmacology study (Clinicaltrials.gov: NCT01978327), running in parallel, which indicated that GSK2657544 had a potentially clinically significant drug–drug interaction (DDI) [14].

## Material and Methods

### Radiopharmaceutical Preparation

[^18^F]GSK2647544 was prepared in a three-step, two-pots synthesis, with intermediate preparation of 4-chloro-3-([^18^F]trifluoromethyl)phenol (Fig. 1). In brief, cyclotron-derived [^18^F]fluoride was first reacted with methyl chlorodifluoroacetate (40 μl) and 4-chloro-3-iodophenyl acetate precursor (9–10 mg) in anhydrous DMF (400 μl) at 150 °C for 15 min. The resulting ester was hydrolysed with tetrabutylammonium hydroxide (1 M in methanol) at 140 °C for 12 min. The formed 4-chloro-3-([^18^F]trifluoromethyl)phenol was transferred to a potassium iodide solution containing 5 g of potassium iodide, 18 ml of water and 2 ml of acetonitrile in order to allow intermediate purification by solid-phase extraction using a C18 SepPak. The SepPak column was washed with 10 ml of a water/acetonitrile solution (90/10, *v*/*v*) upon which excess water was removed using a stream of argon for 2 min. 4-Chloro-3-([^18^F]trifluoromethyl)phenol was released from the SepPak using 4.5 ml of anhydrous diethyl ether. Any trace of water remaining was removed by passing the ether through an online sodium sulphate cartridge and a 4 Å molecular sieve cartridge into a second reactor. The diethyl ether was evaporated at 70 °C under a stream of helium, before being reacted with a bromide-containing precursor (2-(4-bromophenethoxy)-5-(pyrimidin-5-ylmethyl)pyrimidin-4(1H)-one) in anhydrous diglyme (500 μl) at 170 °C for 25 min in the presence of caesium carbonate (48–52 mg) and copper iodide (7–8 mg) to form [^18^F]GSK2647544.

Purification of [^18^F]GSK2647544 was achieved after dilution with 3.5 ml of mobile phase prior to injection onto a reverse-phase high-performance liquid chromatography system (HPLC; Column: Agilent ZORBAX SB C18, 250 × 9.4 mm, 5 μm, Mobile phase: ammonium formate 50 mM pH 4/acetonitrile [50/50, *v*/*v*], flow: 8 ml/min, wavelength: 254 nm). The fraction containing the product was collected and HPLC solvent was removed by means of SepPak reformulation approach to give a product for intravenous (iv) administration in 11 mL of 0.9 % saline containing ethanol (to a maximum of 14 % *v*/*v*) and hydroxypropyl-β-cyclodextrin (to a maximum of 20 % *w*/*w*). The resulting formulated solution was filtered through a 0.2 μm sterile filter (Millipore Millex GV, 0.22 μm, 33 mm) into its final sterile container.

Quality control methods for clinical batches of [^18^F]GSK2647544 were developed in accordance with the European Pharmacopoeia guidelines [15]: residual organic solvents (Ph. Eur. 5.4), pH (Ph. Eur. 2.2.3), pyrogen test (Limulus Amebocyte Lysate (LAL) chromogenic kinetic method, Ph. Eur. 2.6.14), filter integrity test (bubble-point measurement, Ph. Eur. 5.1.1) and sterility test (Ph. Eur. 2.6.1). Other tests included visual appearance, radionuclidic identity and purity.

Radiochemical purity, specific activity and identity were determined by analytical HPLC (Column: Agilent SB-Phenyl [3.5 μm, 4.6 × 100 mm], mobile phase: water [0.2%TFA]/methanol {30/70, *v*/*v*}, flow: 1 ml/min, wavelength: 235 nm, *T* = 50 °C).

### Study Design

This was an open label, non-randomised study to investigate the brain penetration of [^18^F]GSK2647544 by PET and the study protocol allowed for up to 8 evaluable subjects to be scanned. The sample size was based on prior experience from similar brain biodistribution studies. All PET and magnetic resonance imaging (MRI) scans were conducted at Imanova Limited (Burlington Danes Building, Imperial College London, Hammersmith Hospital, Du Cane Road, London W12 0NN, UK) and healthy male subjects were recruited by Hammersmith Medicines Research (HMR, Cumberland Avenue, London, NW10 7EW, UK).

### Subjects

This study was conducted in accordance with the International Conference on Harmonisation of Technical Requirements for Registration of Pharmaceuticals for Human Use (ICH) Good Clinical Practice (GCP) and all applicable subject privacy requirements, and, the ethical principles that are outlined in the Declaration of Helsinki 2008. The original and amended protocols were reviewed and approved by National Research Ethics Service of the UK National Health Service. The study was monitored in accordance with ICH E6, Section 5.18. Written informed consent was obtained from each subject in the study prior to the performance of any study-specific procedures.

Healthy male subjects between 30 and 55 years were recruited for the study, with a body weight of ≥50 kg and body mass index (BMI) within the range 19.0 to 29.0 kg/m^2^ (inclusive). Eligibility was also based on no abnormalities in past medical history, physical examination, vital signs, clinical laboratory parameters and electrocardiography (ECG). See Supplementary Materials for full details of eligibility criteria.

After confirmation that a subject met all eligibility criteria, a structural MRI scan was performed at Imanova Limited to provide a T1-weighted image for co-registration with their PET data. High-resolution (HR) 3D volumetric MRI scans (MPRAGE sequence: TI = 900 ms, TR = 3000 ms, TE = 3.66 ms, flip angle = 9°, voxel size = 1 mm^3^, 160 slices) were acquired for this purpose on a 3 T Tim Trio MRI scanner system (Siemens Healthcare, Erlangen Germany).

### Imaging Procedure

On the day of the PET study, subjects were prepared for the PET scans and received an oral dose of GSK2647544 (100 mg) 2 h before administration of [^18^F]GSK2647544 and the start of the PET scan. Based on the FTIH data [11], this would result in acute blood exposures that were estimated to be within the range required for future therapeutic studies and would help ensure that the measured brain penetration of [^18^F]GSK2647544 was more reflective of this setting.

Subjects were positioned in the PET scanner, after the insertion of a radial artery cannula under local anaesthesia and a venous cannula in an antecubital or forearm vein. The subject’s head was positioned comfortably with soft restraints used to reduce head motion during data acquisition. Dynamic PET data were acquired on one of two Siemens Biograph 6 PET/CT scanners at Imanova (Siemens Healthcare, Erlangen, Germany). A low‐dose CT scan was performed on each subject immediately before each PET scan in order to estimate attenuation. Following iv bolus injection of the radiotracer, dynamic emission data were acquired for 120 min (frame durations: 8 × 15 s, 3 × 60 s, 5 × 120 s, 5 × 300 s, 5 × 600 s). The dynamic images were reconstructed using Fourier rebinning and a 2D filtered discrete inverse Fourier transform algorithm with 5 mm isotropic Gaussian filter on a 128 × 128 matrix with 2.6 zoom giving 2 mm isotropic voxels. Corrections were applied for attenuation, randoms and scatter.

Sampling of radial arterial blood for radioactivity and radioactive metabolites was performed throughout the PET scan, with continuous 5 ml/min for 15 min and discrete sampling from 5 to 120 min post-injection. Whole-blood and plasma radioactivity levels were measured using a Wallac 1470 Wizard gamma counter (Perkin Elmer, Seer Green, UK).

### Metabolite Analysis

Arterial plasma samples for radioactive metabolite analysis were collected at 5, 10, 20, 30, 50, 70, 90 and 120 min post administration of [^18^F]GSK2647544 and were further processed by HPLC using the so-called ‘Hilton’ method [16] to determine the fraction of radioactivity corresponding to the intact parent. Plasma samples (0.5–1 ml) were treated with urea (8 M) to eliminate plasma protein binding, diluted with an equivalent volume of water and filtered through a PTFE membrane. The HPLC consisted of an Agilent 1200 series system (Agilent, Santa Clara, US), including an isocratic pump, a binary pump, a UV detector, a fraction collector and four automated 6-port valves. The capture column was a Biotrap 500MS Column (20 × 4.0 mm, 5 μm) and the analytical column was an Agilent Zorbax Eclipse XDB-C18 (150 × 4.6 mm, 5 μm). The isocratic pump was run at a flow of 2 ml/min using a 5 % acetonitrile in water solution. The binary pump eluent was a mixture of 25 mM pH 3 ammonium formate buffer and acetonitrile. The binary pump was run at a flow of 2 ml/min with following gradient: 5 % acetonitrile from 0 to 4 min, 5 to 95 % acetonitrile from 4 to 7 min, 95 % acetonitrile from 7 to 10 min. UV signal was recorded at 330 nm and fractions were collected every 20 s for gamma counting.

### Free Fraction Measurement

The non-protein bound fraction of [^18^F]GSK2647544 in arterial plasma (*fp*) was quantified using the following methodology. An aliquot of [^18^F]GSK2647544 was added to approximately 1 ml of plasma collected from the subject prior to tracer administration or to 1 ml Tris buffer (pH = 7.4, Sigma). Triplicate 50 μl aliquots of spiked plasma and Tris buffer were prepared for gamma counting using an automated gamma counter (Perkin Elmer, Massachusetts, US). Subsequently, triplicate 200 μl aliquots of spiked plasma and Tris buffer were pipetted into ultrafiltration units (Amicon Ultra-0.5 ml centrifugal filters, Merck Millipore) pre-treated with a 5 % TWEEN 80 solution. Filters were centrifuged at room temperature for 15 min at 10,000 rpm. At the end of centrifugation, 50 μl plasma and Tris ultrafiltrate were counted using the automated gamma counter and *fp* was calculated as the ratio of ultrafiltrate to total activity concentrations corrected for non-specific filter binding. There was no evidence of [^18^F]GSK2647544 being metabolised during this procedure.

### Imaging Data Analysis

Dynamic PET emission and associated structural MRI images were analysed using MIAKAT^TM^, a software developed by Imanova Limited [17]. Dynamic PET emission data was corrected for motion and registered to the structural T1 MRI image. The Imanova Limited neuroanatomical atlas was non‐linearly deformed into the individual’s space in order to generate a personalised anatomical parcellation of the brain. Whole brain (primary endpoint), global grey matter, global white matter, cortex, thalamus and subcortical grey matter were analysed as regions of interest (ROIs) The warped ROIs were applied to the dynamic emission data to generate regional time-activity curves (TACs). Compartmental model analysis was investigated to derive partition coefficient (V_T_) for GSK2647544 for the whole brain as well as for the ROIs. A fixed blood volume correction (5 %) was included in the One Tissue Compartment Model that was selected to derive V_T_.

The single passage extraction fraction (E) for [^18^F]GSK2647544 was calculated as follows: *E* = K_1_/F, where K_1_ is average rate constant for transfer of GSK2647544 from arterial plasma to tissue and F is the brain perfusion, with a value of 0.6 ml/min/cm^3^ [18].

### Bioanalysis of GSK2647544 Following Oral Dosing

Blood samples for the analysis of GSK2647544 plasma concentration were collected at 15, 30, 60, 120 and 240 min post oral dosing into EDTA-K3 tubes, mixed by inversion and immediately cooled to 2–4 °C. Within 50 min of collection, the samples were centrifuged at 1500 g for 10 min at ~4 °C in a swing bucket centrifuge. Plasma was harvested into uniquely labelled polypropylene screw-cap storage tubes, frozen at −20 °C and shipped on dry ice to GlaxosmithKline (Ware, UK) to determine the plasma concentrations of GSK2647544 using a validated analytical method based on protein precipitation, followed by HPLC/MS/MS analysis [GSK method on file].

Plasma concentration-time data was analysed using WinNonlin Professional Edition (Pharsight Corporation, Mountain View, CA). Calculations were based on the actual sampling times recorded during the study. From the plasma concentration-time data, the maximum observed plasma concentration (C_max_) and time to C_max_ (T_max_) were determined.

### Exploratory Analysis for Estimating Brain Lp-PLA_2_ Enzyme Activity Inhibition

Available clinical data indicated that there is a direct E_max_-type relationship between plasma Lp-PLA_2_ enzyme activity inhibition and GSK2647544 plasma concentration [11, 14]. Therefore, such a relationship was assumed between brain Lp-PLA_2_ enzyme activity inhibition and GSK2647544 brain concentration. The percentage brain Lp-PLA_2_ activity inhibition was calculated using the following equation:

$$ \%\ \mathrm{LpPLA}2\ \mathrm{inhibition}=\frac{{\mathrm{C}}_{\mathrm{brain},\mathrm{f}\mathrm{u}}}{{\mathrm{C}}_{\mathrm{brain},\mathrm{f}\mathrm{u}}+\mathrm{I}\mathrm{C}5{0}_{\mathrm{brain},\mathrm{f}\mathrm{u}}}\times 100\% $$

In which, C_brain,fu_ was the free drug concentration of GSK2647544 in the brain, IC50_brain,fu_ was the free drug potency of GSK2647544 in the brain. The IC50_brain,fu_ (0.008 nM) for GSK2647544 was determined using post-mortem human brain tissue [GSK data on file]. The C_brain,fu_ was calculated as: C_plasma_ × B : P × V_T_ × fu_brain_, in which, C_plasma_ was the plasma concentration, B:P was the blood-to-plasma distribution ratio of GSK2647544 (0.606) [GSK data on file], fu_brain_ was the free fraction of GSK2647544 in the brain (0.07 %) [GSK data on file]. For a given bid dose of GSK2647544, C_plasma_ at trough could be predicted based on the PK model derived from the previous clinical studies [11, 14].

## Results

### Brain Biodistribution of GSK2647544

[^18^F]GSK2647544 was successfully prepared using the method described in sufficient yield to support the clinical study objectives (624 ± 192 MBq, 1.0 ± 0.3 % radiochemical yield, 0.5 ± 0.1 GBq/μmol, *n* = 8). The radiochemical purity of [^18^F]GSK2647544, based on analytical radio-HPLC, was recorded to be 100 % for all batches used (*n* = 8) as no other radioactive peaks could be observed. [^18^F]GSK2647544 co-eluted with authentic reference as measured by analytical HPLC, confirming identity. Relatively high mass was observed for the final product suggesting inherent isotopic dilution as a result of the chemical process. These results are in agreement with previous findings [12].

Four male subjects who had an average age of 37 years (age range 34–42) were evaluated in the study. Two hours after oral dosing with unlabelled GSK2647544 (100 mg), the radiolabelled drug was delivered as a slow bolus iv infusion, with an average injected activity of 106 ± 47 MBq and an average injected mass of 179 ± 55 μg. PET dynamic data were successfully acquired from all four subjects.

**Fig. 2:**
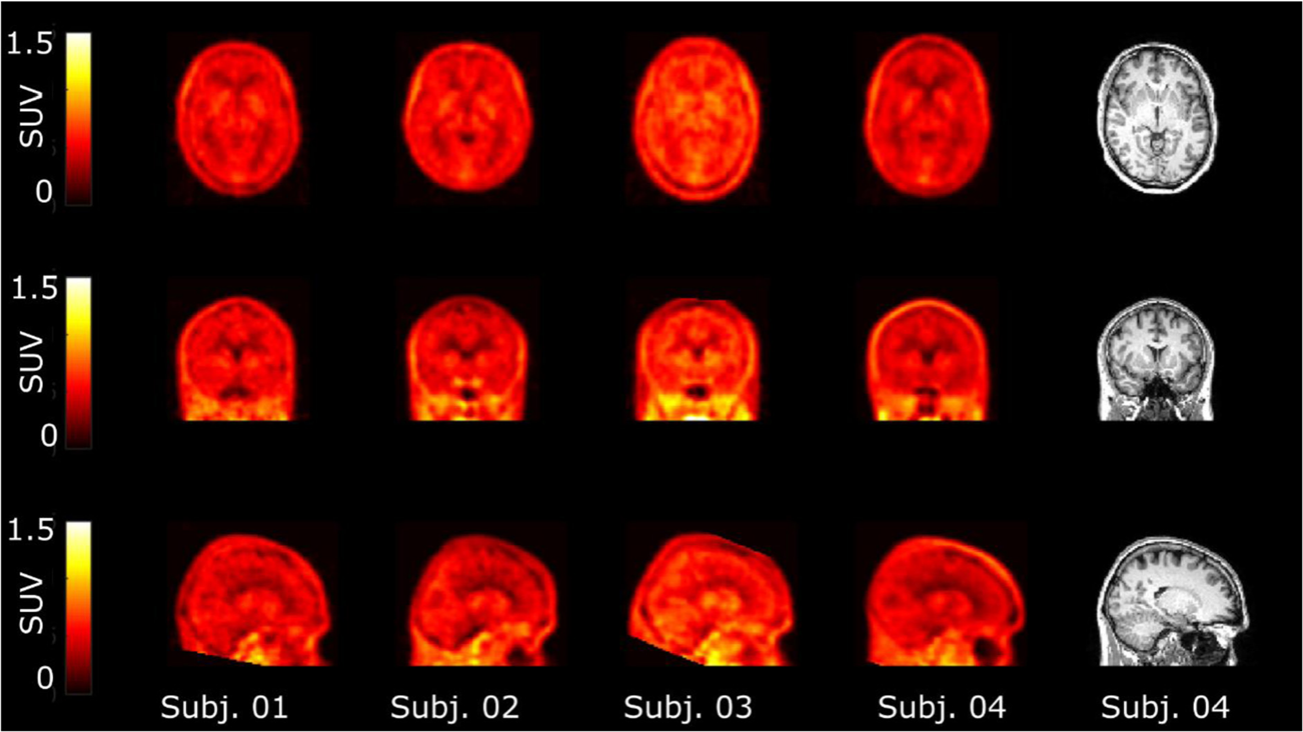
Images showing the summed radioactivity (60–90 min) in the brain of [^18^F]GSK2647544 (*left four panels*) with T1 weighted structural MRI scan show for subject 4 (*right panel*). All four subjects received a 100 mg of unlabelled GSK2647544 followed 2 h later by a bolus injection of [^18^F]GSK2647544 (injected dose: 106 ± 47 MBq). The uptake is expressed standardised uptake values (SUV).

**Fig. 3:**
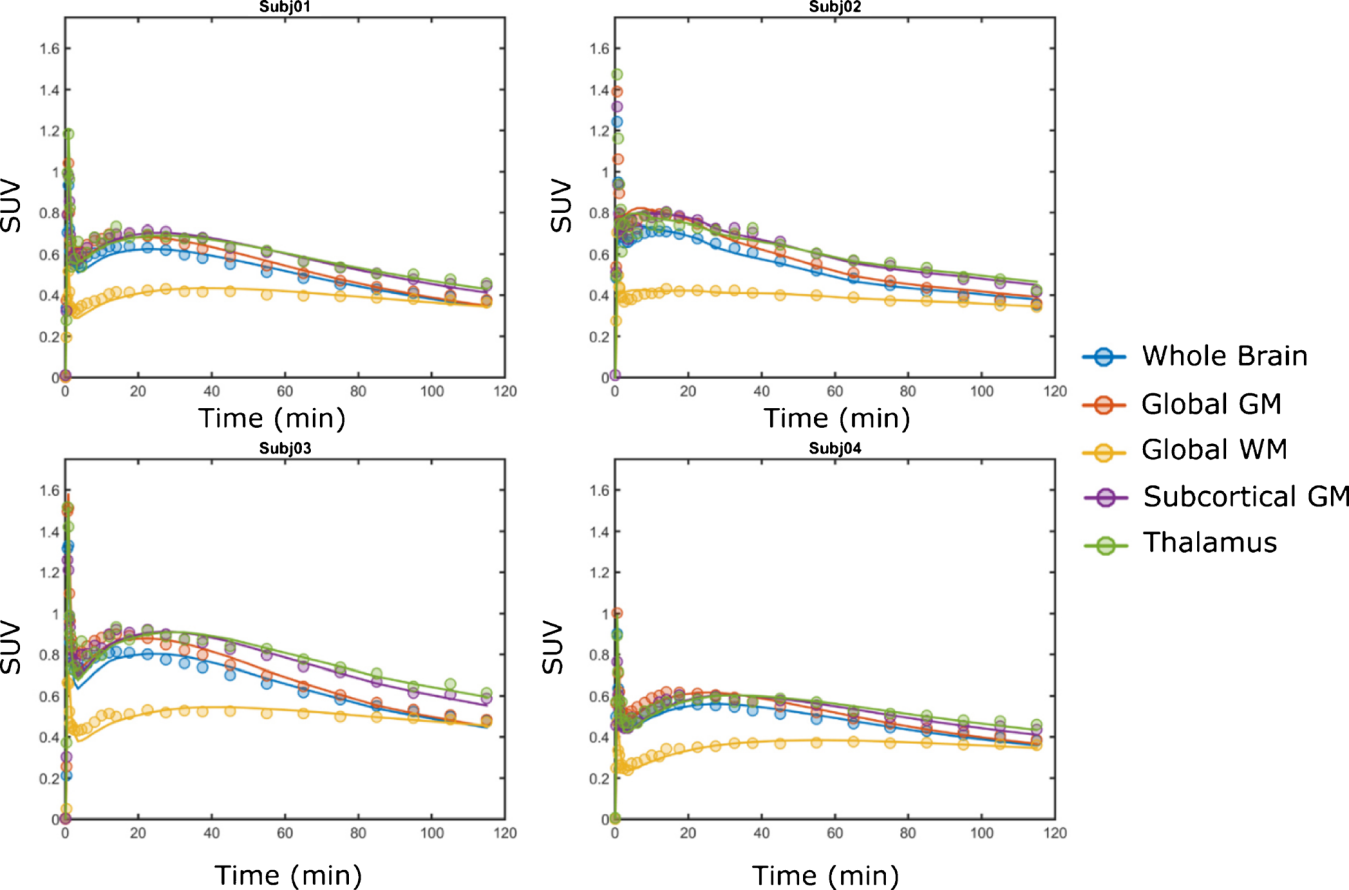
Regional [^18^F]GSK2647544 time-activity curves for each of the subjects. *GM* grey matter, *WM* white matter.

**Fig. 4:**
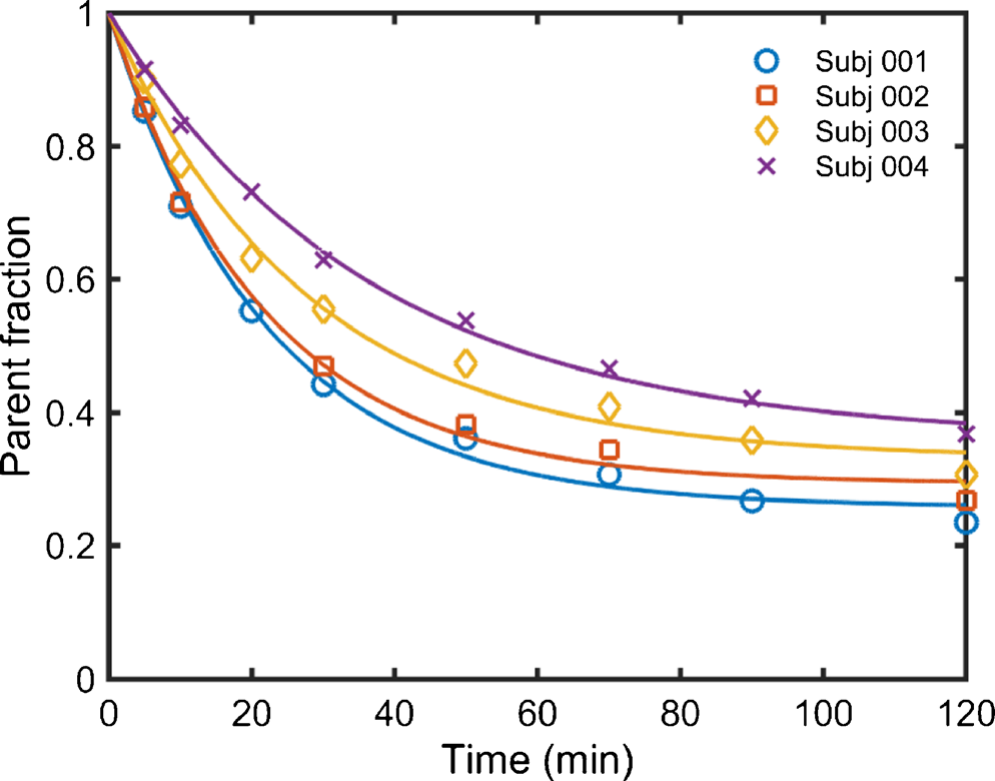
Time course of the parent [^18^F]GSK2647544 fraction in arterial plasma over the duration of the PET scan.

Visual inspection of the PET data indicated a consistent entry of [^18^F]GSK2647544 in the brain and a relatively homogenous distribution of radioactivity across the brain (Fig. 2). The lack of significant regional heterogeneity was confirmed by the inspection of the regional TACs for [^18^F]GSK2647544, although the regional SUVs of subject 4 were generally lower than those of the other subjects (Fig. 3). Analysis of arterial blood and plasma samples allowed the generation of a metabolite corrected arterial plasma input function to enable kinetic analysis of the dynamic image data. Metabolism of GSK2647544 was relatively consistent across the subjects, with 20–40 % of the parent compound present after 120 min (Fig. 4; Supplementary Figure). The presence of a more lipophilic radiometabolite eluting prior to the parent was consistent across scans and was not seen during preclinical method development. While it cannot be formally excluded that the radiometabolite may cross the BBB, good fits were achieved for the kinetic analysis using the metabolite corrected arterial input function, indicating that any contribution of lipophilic metabolites was likely to be marginal.

A one tissue compartmental model (1TCM) provided the most parsimonious description of the data based on the use of a quantitative metric, Akaike information criterion [19]. Application of the 1TCM produced consistent estimates of the model parameters with values reported for V_T_ and K_1_ (Table 1). The primary outcome measure, whole brain V_T_ for [^18^F]GSK2647544, was estimated to be 0.56 (95 % CI, 0.41–0.72). The low variability (<20 %) of the V_T_ values across all regions was consistent with the visual inspection of the images and the regional TACs and supported the view that [^18^F]GSK2647544, when dosed with 100 mg of unlabelled drug, was homogenously distributed throughout the brain. Using an average K_1_ value for the whole brain of 0.0101 ml/gm/min (Table 1), the single passage extraction fraction (E) for [^18^F]GSK2647544 was estimated to be ~2 %.

**Table 1:** Summary of V_T_ and K_1_ values for all 4 subjects across whole brain and ROIs defined in the study

	Volume of distribution V_T_		Rate constant K_1_ (mL/gm/min)	
Brain region	Mean (95 % CI)	Standard deviation	Mean (95 % CI)	Standard deviation
Whole brain	0.56 (0.41,0.72)	0.098	0.0101 (0.0086,0.0117)	0.00097
Cortex	0.52 (0.37,0.66)	0.092	0.0087 (0.0071,0.0103)	0.00100
Global grey matter	0.52 (0.40,0.63)	0.073	0.0099 (0.0093,0.0105)	0.00039
Global white matter	0.50 (0.39,0.61)	0.067	0.0094 (0.0084,0.0105)	0.00066
Subcortical grey matter	0.53 (0.42,0.65)	0.070	0.0117 (0.0104,0.0130)	0.00082
Thalamus	0.54 (0.38,0.71)	0.104	0.0096 (0.0077,0.0114)	0.00116

*V*_*T*_ volume of distribution for the total radioligand in tissue, *K*_*1*_ rate constant for transfer from arterial plasma to tissue

PK analysis of the oral dose of unlabelled GSK2647544 provided estimates of C_max_ (354.0 ng/ml, coefficient variation of 19.1 %) and T_max_ (median 1.4 h, range 1.02 to 6.38 h).

Exploratory modelling suggested that a twice-daily dose of 102 mg, at steady state, would provide ~80 % trough inhibition of brain Lp-PLA_2_ activity.

### Subject Safety

The doses of GSK2647544 administered in this study were well tolerated. All four subjects enrolled in the study completed the protocol and there were no serious adverse events (SAEs), no variation of vital signs and ECG measurements and no clinically significant out of range safety lab results. All the adverse events (AEs) reported during this study were transient and of mild to moderate intensity (Table 2).

**Table 2:** Summary of all adverse events for all four subjects

Total adverse events (*n*)	*N* = 4 subjects
	*n* (%)
Any AE	3 (75)
Any AE related to investigational product	0
Headache	1 (25)
Paraesthesia	1 (25)
Upper respiratory tract infection	1 (25)
Acne	1 (25)

## Discussion

The ability to inhibit brain Lp-PLA_2_, in addition to blood Lp-PLA_2_, is a potentially important feature of GSK2647544. *In vivo* imaging using PET and radiolabelled [^18^F]GSK2647544 was used to explore brain exposure in humans through measurement of the whole brain PET volume of distribution, V_T_, which was the primary outcome measure for the study. The measured V_T_ for [^18^F]GSK2647544 was 0.56 (95 % CI, 0.41–0.72) in the presence of the unlabelled GSK2647544 (100 mg), indicating that the drug is able to enter the brain. Visual inspection of the scan data (Fig. 1) and comparison of the regional TACs (Fig. 2) indicated that the distribution of the radiolabelled drug was broadly homogenous (Table 1). The generally lower regional SUVs for subject 4 were not readily explained by the comparison of the respective PK parameters from each subject (Supplementary Table) suggesting that other factors are likely involved. The secondary PK endpoints (C_max_ and T_max_) were of broadly similar magnitude to that found in the other clinical studies of GSK2647544 [11, 14]. The doses of GSK2647544 administered in this study were well tolerated.

The data from the current study broadly supported the preclinical investigations of the brain penetration of GSK2647544, where blood-to-brain (Kp) ratios of between 0.4 and 0.75 were observed in mice and rats [GSK data on file] (Table 3). Comparison of additional *in vitro* data suggests that GSK2647544 is highly protein bound across species, reflected in the low plasma free fraction (*f*_P_ *)* values, and that the brain free fraction (*f*_ND_) generally appeared slightly higher. Given the additional observations that GSK2647544 was not a Pgp substrate and that its passive permeability across a cellular monolayer model was not limiting [GSK data on file], the data from the biodistribution study was broadly consistent with the preclinical findings. As part of a dosimetry study performed in Sprague-Dawley rats with [^18^F]GSK2647544, a substudy was undertaken to examine the brain penetration of the drug, which found the blood corrected SUV to be ~0.3 [GSK data on file]. No further investigations were performed on the apparently lower overall brain penetration of GSK2647544 observed in this study, and the data did not change the overall design of the clinical PET study. A degree of caution needs to be applied in the cross-comparison and interpretation of the preclinical and clinical data as a number of methodological differences exist across the studies (e.g., route of administration, doses received, observational periods and modelling/data analysis methods).

The preparation of a suitable PET labelled homologue of GSK2647544 provided a significant challenge given the limited availability of functional groups amenable to introduction of the two principal PET isotopes carbon-11 (*T*_1/2_ = 20.4 min) or fluorine-18 (*T*_1/2_ = 109.8 min). A novel chemical pathway [12] provided a potential means to introduce F-18 into the trifluoromethyl moiety of GSK2647544. A multi-step approach, starting from a small initial building block to allow introduction of fluorine-18, had to be designed, as direct labelling of the trifluoromethyl group did not work. It should be noted as well that due to very high retention of the radiotracer on the sterilising filter, hydroxypropyl-β–cyclodextrin was successfully used as an additive in the formulation of the sterile [^18^F]GSK2647544 concentrate solution for injection. To our knowledge, this additive has not been applied in PET formulations to date, which may in part be due to limited availability of a pharmaceutical grade product.

**Table 3:** Listing of available parameters for preclinical species and humans. Free fraction data was generated using equilibrium dialysis methods and for *f*_*ND*_ used brain homogenates. Human tissues were sourced from the Brain and Body Donation Program at Banner Sun Health Research Institute (Arizona, US) and had ethical approval for research use as part of the material transfer agreement

	Free fraction, plasma (*fp*) mean ± SD	Free fraction, brain (*f*_*ND*_) mean ± SD	Kp/V_T_^1^
Mouse	Not determined	0.00068 ± 0.00002, *n* = 3	0.41^a^
Rat	0.0005 ± 0.0001, *n* = 3	0.00071 ± 0.00002, *n* = 3	0.75^b^, 0.64^c^
Human	0.0004 ± 0.00003, *n* = 3	0.00068 ± 0.00015, *n* = 6	0.56^1^

^a^2 mg/kg, intraperitonal, AUC_0–4h_

^b^2 mg/kg, oral, AUC_0–7h_

^c^10 mg/kg, oral, AUC_0–7h_

The current study provides a demonstration of the utility of this new radiochemical approach and provides easy access to F-18 radiolabelled trifluoromethyl groups, frequently used to enhance pharmacokinetic properties. This reaction involves only commercially available reagents and readily accessible iodinated precursors. The generation in situ of the [^18^F]CF3Cu reagent involves the formation of a carbene intermediate, which explains the observed low specific activity values. As a result, this technology could not be applied where low mass is required, e.g., brain neuroreceptor occupancy studies, but, given the high prevalence of the trifluoromethyl group, it is highly relevant for labelling candidate drug molecules to understand their CNS biodistribution and in combination with described approach for GSK2647544 provides a means to estimate target occupancy in the absence of a specific tool compound. An additional potential limitation of this technique is the low radiochemical yield which may make it unsuitable in situations where high volumes of scans need to be performed (e.g., as a diagnostic tool) but in the current situation the yield was fit-for-purpose. Nevertheless, relatively straightforward PET biodistribution studies, using this labelling methodology, have the potential to be utilised early in clinical development and can add valuable information for early compound progression decisions.

Due to the decision to terminate the PET study, due to the separate DDI finding [14], an amended protocol that would have permitted the administration of [^18^F]GSK2647544 in the absence of the 100 mg dose of unlabelled drug was not initiated. As the observed yield of [^18^F]GSK2647544 was ~5- to 10-fold higher than anticipated during the clinical manufacture, this provided the opportunity to determine the whole brain V_T_ of [^18^F]GSK2647544 under more tracer-like conditions (i.e., in the absence of the loading dose of unlabelled drug). This approach would have facilitated comparison with the currently determined whole brain V_T_ values to explore whether the mode of entry of the drug was potentially through passive diffusion or subject to transporter effects, thereby providing valuable reverse-translational validation of the preclinical models used to measure brain penetration. Therefore, this aspect was considered further through the following exploratory analysis.

Molecules entering the brain by passive diffusion are expected to reach similar free concentrations at equilibrium. With this in mind, it can be shown that the ratio of the *in vitro* derived plasma (f_P_) and brain (f_ND_) free fractions will be equal to the equilibrium partition coefficient for the drug at equilibrium [20]. As the PET V_T_, for regions with minimal displaceable binding, can be shown to be equivalent to the equilibrium partition coefficient, brain entry by passive diffusion can be assumed if V_T_ ≅ f_P_/f_ND_ [20]. The data from this study provide little evidence for a displaceable binding component with [^18^F]GSK2647544 due to the lack of regional heterogeneity and the high dose of GSK2647544 (100 mg) pre‐administered before the scan. For GSK2647544, based on an equilibrium dialysis method using post-mortem human brain homogenates, f_P_ and f_ND_ were estimated to be 0.0004 and 0.00068 [GSK data on file] (Table 3), respectively. A comparison of the mean value of V_T_ (0.56, Table 2) with f_P_/f_ND_ (0.59, i.e., 0.0004/0.00068) suggested that due to their numerical similarity, the entry of GSK2647544 was consistent with a passive diffusion mechanism and therefore, it may be expected that at equilibrium, the free brain concentration of GSK2647544 will be equivalent to the free plasma concentration (i.e., f_ND_ ≅ f_P_).

There are limited reports relating to the molecular imaging of lipid-related metabolism in the brain, one such example is the C-11 labelled form of arachidonic acid (AA) that has been used in man to investigate dopaminergic neurotransmission [21] and neuroinflammation [22]. AA is a polyunsaturated fatty acid that is found incorporated high concentrations in brain phospholipids and the metabolism of AA and its oxidatively modified form, F2-isoprostane, has been linked with cPLA_2_ and Lp-PLA_2_, respectively [23, 24]. Although Lp-PLA_2_ has been implicated in F2-isoprostane metabolism, the evidence is mixed and may indicate a more significant role for other phospholipases [24, 25]. The further evaluation of potential Lp-PLA_2_ tracers and development of further molecular imaging agents to probe brain lipid metabolism [26] can only serve to improve our understanding of disease-related changes in these important pathways.

## Conclusion

In conclusion, a novel synthetic method was used to generate [^18^F]GSK2647544 and this was successfully applied in the current PET biodistribution study. The findings from this study indicate that GSK2647544 is able to cross the blood brain barrier and enter the human brain. An exploratory analysis of the data indicated that a dose of 102 mg, twice daily at steady state would be sufficient to reach, at trough, ~80 % brain Lp-PLA_2_ inhibition.

## Electronic Supplementary Material

Below is the link to the electronic supplementary material.

ESM 1 **Supplementary Figure**. Top. Chromatogram of [18 F]GSK2647544 reference sample in plasma. Bottom. Average chromatogram of plasma samples from the 4 subjects analysed for one specific timepoint (70 min post-injection). Fractions were analysed post-HPLC using a gamma counter. **Supplementary Table**. Individual PK parameter estimates. (PDF 111 kb)
