# Supplementary material for: Investigation of the Brain Biodistribution of the Lipoprotein-Associated Phospholipase A2 (Lp-PLA2) Inhibitor [18F]GSK2647544 in Healthy Male Subjects
Source: Mol Imaging Biol. 2016 Jul 11;19(1):153–61. doi: 10.1007/s11307-016-0982-5 (PMC5209404; doi:10.1007/s11307-016-0982-5)
Supplement: Supplementary file 1 — Supplementary Figure. Top. Chromatogram of [18 F]GSK2647544 reference sample in plasma. Bottom. Average chromatogram of plasma samples from the 4 subjects analysed for one specific timepoint (70 min post-injection). Fractions were analysed post-HPLC using a gamma counter. Supplementary Table. Individual PK parameter estimates. (PDF 111 kb) [file 11307_2016_982_MOESM1_ESM.pdf]

**Electronic Supplementary Material**

# Investigation of the Brain Biodistribution of the Lipoprotein aAssociated Phospholipase $A_2$ (Lp-$PLA_2$) Inhibitor [$^{18}$F]GSK2647544 in Healthy Male Subjects

**Journal: Molecular Imaging and Biology**

Mickael Huiban[1], Christopher Coello[1], Kai Wu[2], Yanmei Xu[3], Yvonne Lewis[1], Andrew P. Brown[4], Mauro Buraglio[5], Chenbing Guan[3], Shaila Shabbir[5], Regan Fong[6], Jan Passchier[1], Eugenii A. Rabiner[1], and Andrew Lockhart[7]

Corresponding author: A. Lockhart, andrew.2.lockhart@gsk.com; Ph+44(0)1223296001; Fax +44(0)1223296002

[1]Imanova Limited, Burlington Danes Building, Imperial College London, Hammersmith Hospital, Du Cane Road, London, W12 0NN, UK

[2]WuXi Clinical Development Service, 19th Floor, Building A, FuXing Plaza, 388 Ma Dang Road, Shanghai China, 200025

[3]GlaxoSmithKline, Neurosciences, 917 Halei Road, Zhangjiang Hi-tech Park, Pudong, Shanghai 201203, China

[4]AstraZeneca UK Limited, Melbourn Science Park, Royston, Herts., SG8 6HB UK

[5]GlaxoSmithKline, Neurosciences, Gunnels Wood Road, Stevenage SG1 2NY, UK

[6]UCB Biosciences Inc, PO Box 110167, Research Triangle Park, North Carolina 27709 USA

[7]GlaxoSmithKline, Neurosciences, Clinical Unit Cambridge, Addenbrooke's Hospital, Cambridge CB2 0GG, UK

**Supplementary materials**

Selection of Study Population

Inclusion Criteria

A subject was eligible for inclusion in this study only if all of the following criteria apply:

1. Healthy as determined by a responsible and experienced physician, based on a medical evaluation including medical history, physical examination, laboratory tests and cardiac monitoring. A subject with a clinical significant abnormality or laboratory parameters significantly outside the reference range for the population being studied were included only if the Investigator and the GSK Medical Monitor agreed that the finding was unlikely to introduce additional risk factors and did not interfere with the study procedures.

2. Male subject between 30 and 55 years of age inclusive, at the time of signing the informed consent.

3. Male subjects agreed to use one of the contraception methods listed in Protocol Section 4.3.1.1. This criterion was followed from the time the first administration of GSK2647544 until completion of the study and for 4 months after dosing.

4. Body weight ≥50 kg and body mass index (BMI) within the range 19.0 to 29.0 kg/m2 (inclusive).

5. Capable of giving written informed consent, which included compliance with the requirements and restrictions listed in the consent form.

6. Adequate collateral flow to the radial and ulnar arteries in both hands as determined by an Allen's test.

Exclusion Criteria

A subject was not eligible for inclusion in this study if any of the following criteria apply:

Criteria Based Upon Medical Histories:

1. A screening ECG with a corrected QT (QTc) value, of<350msec or >450msec (triplicate ECGs) and/or a PR interval outside the range 120 to 220msec (triplicate ECGs) or an ECG that was not suitable for QT measurements (e.g. poorly defined termination of the T-wave).

2. Pulse rate <45 or >90 beats per minute (BPM) OR a systolic BP >140 or <90 OR a diastolic BP >90 or <60.

3. History of long QT syndrome (personal or family) or other cardiac conduction disorder, a history of sudden unexplained death or unexplained syncope in a first degree relative or other clinically significant cardiac disease.

4. Subjects with current or past diagnosis of cardiovascular disease including but not limited to hypertension, cardiac arrhythmias and/or risk factors for coronary artery disease.

5. Current or recent (within one year) gastrointestinal disease; a history of mal-absorption, oesophageal reflux, irritable bowel syndrome; frequent (more than once a week) occurrence of heartburn; or any surgical intervention (e.g., cholecysectomy) which would be expected to influence the absorption of drugs.

6. History of asthma, anaphylaxis or anaphalactoid reactions, severe allergic responses.

7. History of hypercoagulable state or history of thrombosis.

8. Subjects who had current renal or history of clinically significant renal abnormalities.

9. History or presence of a neurological or psychiatric diagnosis (not limited to but including for example, stroke, traumatic brain injury, epilepsy, space occupying lesions, multiple sclerosis, Parkinson's disease, vascular dementia, transient ischemic attack, schizophrenia, major depression etc) that in the opinion of the investigator influenced the outcome or analysis of the scan results.

10. Subject was mentally or legally incapacitated.

11. Use of prescription or non-prescription drugs, including vitamins, herbal and dietary supplements (including St John's Wort) within 7 days (or 14 days if the drug was a potential enzyme inducer) or 5 half-lives (whichever was longer) prior to the first dose of study medication, unless in the opinion of the Investigator and GSK Medical Monitor, the medication did not interfere with the study procedures or compromise subject safety.

Criteria Based Upon Diagnostic Assessments:

1. A positive pre-study drug/alcohol screen. A minimum list of drugs that were screened included amphetamines, barbiturates, cocaine, opiates, cannabinoids and benzodiazepines.

2. Urinary cotinine levels indicative of smoking or history or regular use of tobacco- or nicotine-containing products within 6 months prior to screening.

3. History of regular alcohol consumption within 6 months of the study defined as: an average weekly intake of >21 units for males or >14 units for females. One unit is equivalent to 8 g of alcohol: a half-pint (~240 mL) of beer, 1 glass (125 mL) of wine or 1 (25 mL) measure of spirits.

4. A positive pre-study Hepatitis B surface antigen (HBsAg) or positive Hepatitis C antibody result within 3 months of screening.

5. A positive test for human immunodeficiency virus (HIV) antibody.

Other Criteria:

1. History of sensitivity to any of the study medications, or components thereof or a history of drug or other allergy that, in the opinion of the investigator or GSK Medical Monitor, contraindicated their participation.

2. The subject had participated in a clinical trial and had received an investigational product (IP) within the following time period prior to the first dosing day in the current study: 3 months, 5 half-lives or twice the duration of the biological effect of the IP (whichever was longer).

3. Exposure to more than three new chemical entities within 12 months prior to the first dosing day.

4. Unwillingness or inability to follow the procedures outlined in the protocol.

5. Previous inclusion in a research and/or medical protocol involving nuclear medicine, PET or radiological investigations with significant radiation burden (a significant radiation burden defined as >10mSv in addition to natural background radiation in the previous 3 years).

6. Worked as a welder, metal worker or machinist.

7. History of, or suffers from, claustrophobia or felt that he was unable to lie still on his back in the PET or MRI scanner for a period of 1-2 h.

8. Presence of a cardiac pacemaker or other electronic device or ferromagnetic metal foreign bodies as assessed by a standard pre-MRI questionnaire.

9. Where participation in the study resulted in donation of blood or blood products in excess of 500 mL within a 56 day period.

Supplementary Figure

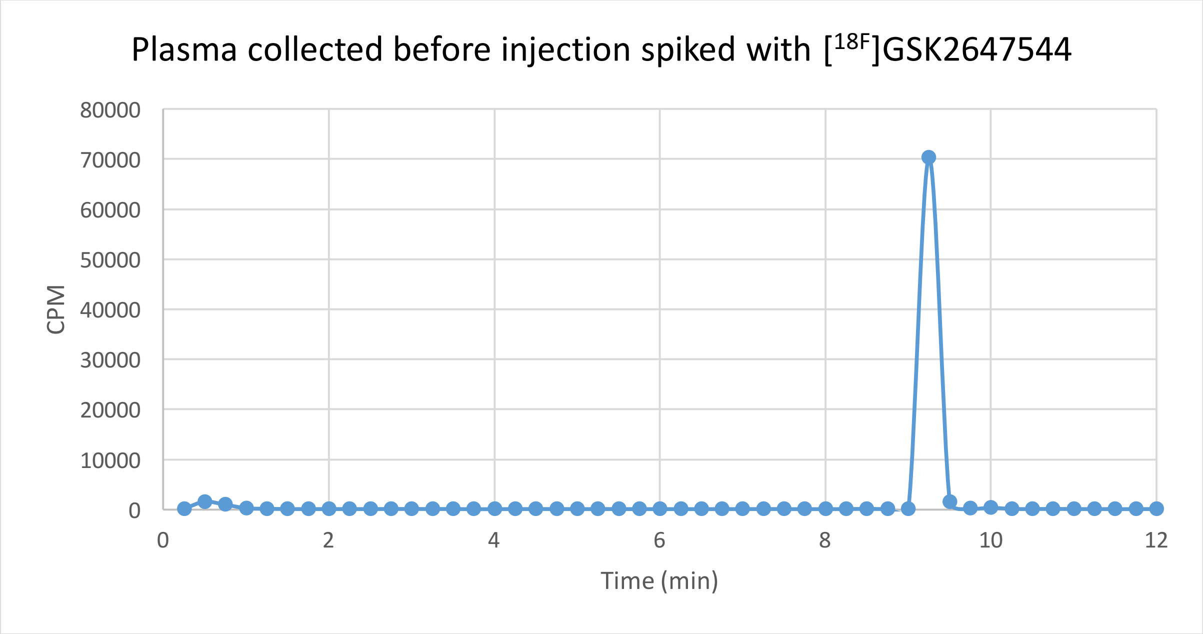


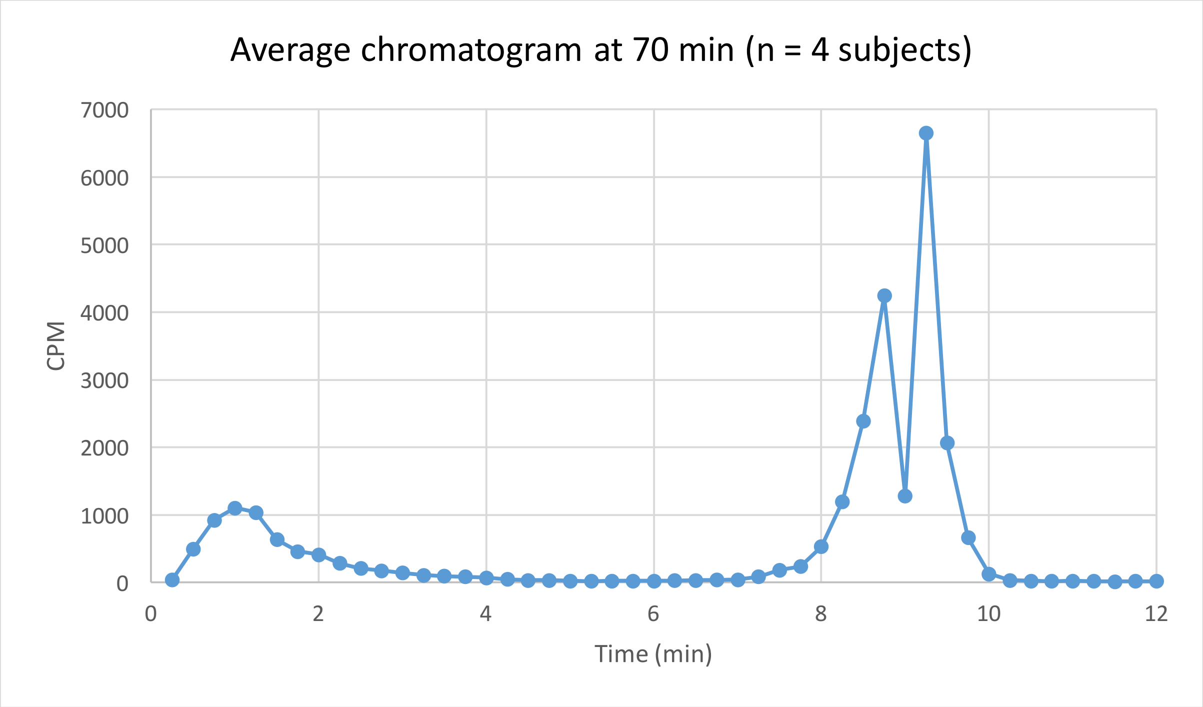


Supplementary Figure. Top. Chromatogram of [18F]GSK2647544 reference sample in plasma. Bottom. Average chromatogram of plasma samples from the 4 subjects analysed for one specific timepoint (70 minutes post-injection). Fractions were analysed post-hplc using a gamma counter.

| Subject ID | $T_{max}$ (h) | $C_{max}$ (ng/mL) |
| --- | --- | --- |
| 1 | 1.03 | 346.91 |
| 2 | 6.38 | 273.06 |
| 3 | 1.02 | 415.20 |
| 4 | 1.85 | 399.16 |

Supplementary Table. Individual PK parameter estimates.
